# Effects of virtual fencing on behavior, cortisol concentrations, feed intake, and milk yield of lactating dairy cows in different grazing systems

**DOI:** 10.1093/jas/skaf363

**Published:** 2025-10-22

**Authors:** Brigitte G C de Bruijn, Eline E A Burgers, Ingrid D E van Dixhoorn, Martine H Bruinenberg

**Affiliations:** Animal Health and Welfare Department, Wageningen Livestock Research, Wageningen, The Netherlands; Animal Nutrition Department, Wageningen Livestock Research, Wageningen, The Netherlands; Animal Health and Welfare Department, Wageningen Livestock Research, Wageningen, The Netherlands; Animal Nutrition Department, Wageningen Livestock Research, Wageningen, The Netherlands

**Keywords:** dairy cattle, pasture management, precision livestock farming, rotational grazing, welfare

## Abstract

Virtual fencing system (VF) is suggested to optimize grazing management on dairy farms. The objective of this study was to evaluate the application of VF for lactating dairy cows in a daily or weekly rotational grazing system and its effect on behavior, cortisol concentrations, feed intake, and milk yield. A completely randomized block design with 64 lactating dairy cows was used with four treatments within a 2 × 2 factorial design. Treatments were: VF with weekly rotation to a new plot (VFW), VF with daily rotation to a new plot (VFD), physical electric fencing (EF) with weekly rotation to a new plot (EFW) and EF with daily rotation to a new plot (EFD). The cows were naïve to VF prior to the study. At night cows were housed indoors separately per treatment and a partial mixed ration was fed. During the day all cows grazed at the pasture. After a 4-d adaptation period on the pasture with EF for all cows, the VF cows were trained over 4 d with the VF (^®^ Nofence, AS, Batnfjordsøra Norway). After the training period, all cows grazed in separate plots per treatment within the specific fencing and grazing system for four weeks (measurement period). Number of auditory and electrical cues, success, success ratio and confidence ratio (which weighs the success ratio against the proportion of auditory cues) were calculated from the VF data. Behavior was continuously recorded by activity sensors (SensOor, Harmelen, the Netherlands) for all cows. Behavioral observations were performed throughout the study. Milk samples were taken on day 2, 6, 9, 13, 16, 20, 27, and 34, and milk cortisol concentrations were analyzed using ELISA. At the start and end of the study hair samples were taken and hair cortisol concentrations were measured. Daily milk production, feed intake indoors and at the pasture were recorded throughout the study. The study showed no differences between cows in VF or EF in behavior, cortisol concentrations in hair and milk, feed intake, and milk yield parameters. Regardless of the grazing system, an increase in the number of successes, success ratio, and confidence ratio was observed for all cows with VF during the measurement period. Cows in VFD had a higher success and confidence ratio than VFW cows, indicating that more frequent shifts of the VF enhanced learning ability and confidence in the VF. In conclusion, VF did not affect behavior, cortisol concentrations, feed intake, and milk yield of lactating dairy cows, irrespective of the grazing system.

## Teaser

Virtual fencing in different rotational grazing systems did not affect behavior, cortisol concentrations, feed intake, and milk yield of lactating dairy cows.

## Introduction

In the Netherlands and other European countries such as Ireland, Sweden, and Belgium, most dairy systems are pasture-based, with fresh grass serving as a major component of the diet of dairy cows (van den Pol-van Dasselaar et al., 2020). In the Netherlands, 73% of all dairy cows had access to grazing in 2024 (CBS, 2024), which is also valued as important by the public (Elgersma, 2012; van den Pol-van Dasselaar et al., 2020). As such, grazing can be considered a vital part of dairy farming. Grazing efficiency can be optimized by creating a match between fresh grass allowance and cow requirements, while minimizing back-grazing of new leaves which would slow pasture regrowth (Hennessy et al., 2015; Klootwijk et al., 2019; Mayel et al., 2021). One way to realize this match could be by applying rotational grazing (Roche et al., 2017), where cows receive a new grazing area every period of time. However, applying rotational grazing can be labor intensive, as fences and cows usually have to be moved manually.

One way to reduce the labor intensity of grazing cows and specifically rotational grazing could be by using a virtual fencing system (VF) (Goliński et al., 2023). With VF, boundaries for grazing cows can be created without the need for a physical fence, facilitating the management of these boundaries. In such a system, animals are usually equipped with a global navigation satellite system (GNSS) collar, and the VF can be delineated via an application. If an animal approaches the VF, the collar delivers auditory cues and, if those are ignored and the animal is about to cross the boundary, an electrical cue is delivered to prevent the animal from exceeding the boundary. Several studies reported a successful application of VF in different rotational grazing systems (Langworthy et al., 2021; Verdon et al., 2021; Grinnell et al., 2025). Uniform utilization of pasture indicated that cows grazed near the VF boundary (Langworthy et al., 2021), and in a 28-d period 90% of cows spent ≤ 0.15% of the time on the pasture outside the VF boundaries (Verdon et al., 2024).

When evaluating new husbandry technologies such as VF, animal welfare should be closely considered and successful learning of the VF is essential. The stress response should be minimal once cows have learned to comply with the cues from the VF, leading to a predictable and controllable grazing environment (Lee et al., 2018). When Fleckvieh heifers followed a 12-day training schedule with different VF boundaries, they were all able to learn to respond to auditory cues to avoid the electrical cue (Hamidi et al., 2024). Moreover, after 10-d grazing in a pasture with physical electric fences (EF) followed by a 3-d training period, the replacement of one EF by VF during a 10-day period did not affect behavior, stress levels as indicated by milk cortisol concentrations or milk yield (Verdon et al., 2021). However, a daily rotation to a new pasture for an extended period was suggested to complicate the learning ability, as cows are challenged to rediscover the location of the virtual boundary every day (Verdon et al., 2021), thereby possibly affecting their behavior or stress levels. Cow welfare in rotational grazing systems was not affected when comparing VF with EF in terms of activity, lying behavior, and milk cortisol concentrations with boundary changes every 7–21 d (Fuchs et al., 2024) or in terms of fecal cortisol metabolite concentrations, herbage selection and body weight gain with boundary changes every 3–4 d (Grinnell et al., 2025). To our knowledge, effects of daily rotational grazing with VF on behavior and stress response have not been investigated yet, although this grazing strategy is frequently used in various countries.

This study aimed to evaluate the application of VF for lactating dairy cows in both a daily and weekly rotational grazing system. We investigated the effect of grazing system (daily and weekly rotation) on the number of auditory cues and electrical cues that cows received from the VF. Moreover, we investigated the combined effect of grazing system and type of fencing (VF or EF) on behavior, cortisol concentrations in hair and milk, feed intake, and milk yield of dairy cows. It was hypothesized that cows that rotated daily to a new pasture had more difficulties in complying with the VF indicated by a higher number of auditory and electrical cues and reduced success compared with cows that were rotated to a new pasture weekly. In addition, it was hypothesized that VF cows did not differ in milk yield, feed intake, and cortisol concentrations in hair and milk compared with EF cows throughout the study.

## Materials and Methods

The established principles of laboratory cow use and the Dutch laws related to cow experiments were adhered to in this study. This study was conducted under the Dutch law on Animal Experiments in accordance with European Union Directive 2010/63 and approved by the Animal Welfare Body of Wageningen Research (Lelystad, Netherlands).

### Cows and study design

This study was conducted over 35 d in the spring of 2024 at the Dairy campus research farm of Wageningen University & Research in Leeuwarden, the Netherlands (53°10′50.7″N, 5°45′25.2″E). Forty-six multiparous Holstein dairy cows entered the study and were selected based on the following criteria: parity ≥2, >100 days in lactation, and experience with grazing but naïve to VF.

The study was conducted using a completely randomized block design with four treatments within a 2 × 2 factorial design. Cows were either grazed with EF or VF and were either rotated daily or weekly to a new plot. This resulted in four treatments with each 16 dairy cows: electric fencing with daily rotation (EFD), electric fencing with weekly rotation (EFW), virtual fencing with daily rotation (VFD) and virtual fencing with weekly rotation (VFW). The cows were blocked in groups of four balanced for parity, lactation stage, milk yield and composition, and body weight. Within each block, cows were randomly assigned to one of the four treatments. The cows in the VF groups were equipped with a VF collar and the cows in the EF groups were equipped with a dummy collar with similar weight as the VF collars.

During the day, cows had access to pasture, while they were housed indoors at night. The study was divided into three periods: 1) a 4-d adaptation period during which all cows grazed together on the same pasture to acclimate to grazing conditions using EF. This was followed by period 2) a 4-d training period, during which VF cows were trained to understand the VF. From the start of the training period, cows remained in their assigned treatment groups and grazed on separate plots. Period 3 was the measurement period, which lasted 4 wk (wk 1–4). During this period, cows were either rotated weekly or daily, depending on the assigned treatment.

### Housing

Cows grazed on pasture A (6.1 ha) and B (7.1 ha) in which a plot was created for each treatment (2 on A, 2 on B). The perimeter fencing of the pastures consisted of EF, while the internal pasture divisions around the fields were either VF or EF, based on the assigned treatment.

**Figure 1.**
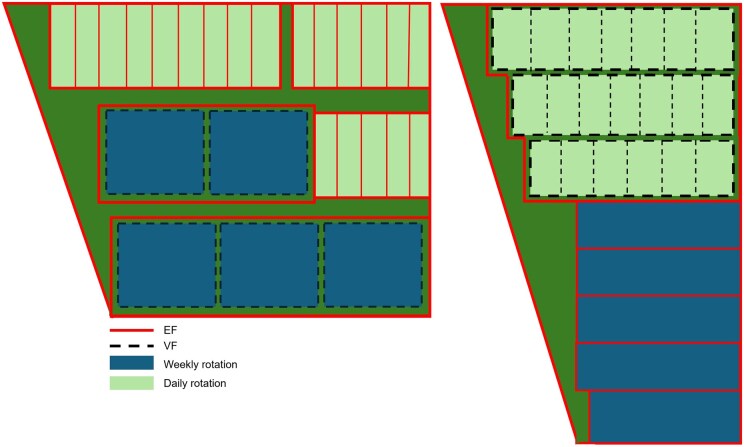
Schematic overview of the pasture design during the measurement period. Red lines indicate physical electric fences (EF) and black dashed lines indicate virtual fences (VF). Blue plots were used for weekly rotational grazing and light green plots were used for daily rotational grazing.

During the adaptation period (1), all 64 cows from all 4 groups grazed together on a plot of 1.43 ha (223 m^2^/cow). During the training period (2), the cows grazed per group on plots of approximately 0.8 ha (500 m^2^/cow). During the measurement period (3), the cows that rotated daily were rotated over a pasture of 0.7 ha. Per day, 0.17 ha per group was available (106 m^2^/cow). At the end of each day, the (virtual) fencing was moved so that each day half of the pasture (0.085 ha) consisted of fresh grass, and the other half consisted of grass from the day before (0.085 ha). Cows in the groups that rotated weekly had access to 0.5 ha (313 m^2^/cow). Every week these cows went to a completely new plot of 0.5 ha. The pasture sizes were based on the assumption of a fresh grass intake of 8 kg dry matter per cow per day (during 10 h of grazing; Klootwijk et al., 2020). A schematic overview of the pasture design during the measurement period can be found in Figure 1.

Cows were milked twice daily at intervals of 11–13 h in a 40-cow rotary milking parlor (Gea Farm Technologies, Deventer, Netherlands). After the morning milking, all cows were moved to their designated pasture (depending on the treatment). After the evening milking, they were returned to the barn where they were housed, and they were kept in separate pens per treatment. All groups had unrestricted access to drinking water, both in the pasture and in the barn. During the night, all cows were provided with a partial mixed ration (PMR) consisting of grass silage, corn silage, soybean meal, dried sugar beet pulp, and lactation minerals at the feed fence in the barn. Additionally, cows received concentrate feed in automated feeders (Manus VC5, DeLaval, Steenwijk, Netherlands), according to their milk yield, and were supplemented with 1 kg of pelleted feed in the milking parlor each day.

### Climate conditions

The Dutch Weather Service shows an annual average temperature of 9.9 °C, an annual precipitation sum of 829 mm and an annual radiation sum of 1,789 h at the weather station in Leeuwarden at 3.6 km for the research facility. At the weather station, in May 2024 the average temperature was 15.3°C, the precipitation sum was 114 mm and the radiation sum was 248 h. In June 2024 the average temperature was 15.0°C, the precipitation sum was 88.6 mm and the radiation sum was 223 h (Royal Netherlands Meteological Institute, 2021, 2024).

### Virtual fencing technology

Commercial VF collars (^®^ Nofence, AS, Batnfjordsøra Norway) were used in this study. The VF consists of a collar with a weight of 1446 g and included a collar unit, rechargeable battery, neck strap, solar panels on both sides, GNSS receiver and a ‘Nofence App’ using 2G and 4G networks to communicate with the collars (NoFence, 2025).

Virtual boundaries were set using the NoFence App for the VF treatments. When a cow approached a set virtual boundary at a distance of 1 meter, the collar gave an auditory cue, which increased in pitch. If the cow did not distance itself from the boundary or continued walking over the boundary, the collar gave an electrical cue after the last (15th) pitch (electrical cue: 0.2 J at 3 kV for 1 s; auditory cue: 82 dB). Ignoring the auditory cues resulted in a max of three consecutive electrical cues. After this, the cow was designated as “escaped” and no further signals were given to the cow, but the location could still be tracked on the App. If the cow re-entered the virtual pasture, the collar returned to normal functionality.

The VF cows were trained with the VF in separate pastures per treatment. In short, during the first day of training one VF boundary was set parallel to one (out of four) of the EF boundaries on a 5-m distance. In this way, the cows grazed in a pasture with three EF boundaries and one VF boundary. On the following days of training, every day one VF boundary was added parallel to an EF boundary on a 5-m distance. On day four, the cows were fully enclosed within the VF. During training, cow responses to auditory and electrical cues were assessed both visually during behavioral observations on day 1 and 4 of training (described later) and by reviewing daily data on the number of auditory and electrical cues received. This evaluation aimed to identify any cows that received an excessive number of electric cues from the collar system. These cows were not identified and hence, all cows entered the measurement period.

### Data collection

#### Virtual fencing data

The VF data including auditory and electrical cues emitted from the collars, escape notifications, routine status updates and GNSS locations were provided by the manufacturer for scientific purposes at the end of the trial. The timestamp, auditory cues and electrical cues were used for analyses. The auditory cues and electrical cues were aggregated per cow per day, and were used to calculate the success (electrical cues—auditory cues; Hamidi et al., 2024). This indicates how well the cow was able to prevent the electrical cue after receiving the auditory cue. Based on the success, the success ratio was calculated (success/total number of auditory cues; Eftang et al., 2022; Hamidi et al., 2024). Lastly, the confidence ratio was calculated by multiplying the success ratio by the success and dividing the result by 20. The confidence ratio reflects the voluntary interactions with the VF and is an indication of the cows’ ability to interact with the boundary without receiving an electrical cue (Hamidi et al., 2024).

#### Cortisol measurements in milk

Milk samples were taken for all cows for eight morning milkings (day 2, 6, 9, 13, 16, 20, 27, and 34) to detect patterns in milk cortisol concentrations over time. Milk samples were collected in 15 mL plastic tubes and frozen at –20°C. All samples were analyzed using a salivary cortisol ELISA kit (Salimetrics LLC., State College, PA, USA) according to the kit’s protocol.

#### Cortisol measurements in hair

Hair samples were taken for all cows 7 d before the start of the study and on the last day of the study. Hair samples of the dominant hair color were taken on the left flank near the spine. An area of 20 × 20 cm was shaved using hair clippers for livestock, and the samples were then stored in the dark at −20°C. The hair samples were submitted to the veterinary laboratory of Royal GD (Deventer, the Netherlands) for analysis. Cortisol concentrations in hair samples were analyzed based on a protocol adapted from previous studies (Koren et al., 2002; Burnett et al., 2014). Briefly, this method was as follows: hair samples were washed three times with isopropanol and subsequently dried in an oven at 37°C during approximately 24 h. Samples were then ground into hair powder using a Retch grinding cup with a 20 mm bead in a Retch beater for 5 min. About 200 mg of hair powder was transferred into glass tubes. Methanol (5 mL) was added to the tubes and mixed with the hair powder. Tubes were then sonicated for 30 min, placed in an oven at 50°C for approximately 24 h and subsequently centrifuged for 10 min. After centrifugation, 4 mL solution of the samples was pipetted into new glass tubes, and the methanol was evaporated using nitrogen. Subsequently, 500 µL of physiological saline solution was added to each tube and the tubes were mixed, placed in an ultrasonic bath for 10 min and mixed again. The cortisol concentration was then analyzed in the sample solutions with an IDS-iSYS Salivary Cortisol test kit (IS-4900) on an IDS-iSYS Multi-Discipline Automated System. Results were recalculated to the cortisol concentration in ng/g hair.

#### Behavior

All cows were equipped with an activity sensor (SensOor, CowManager, Harmelen, the Netherlands) attached to the left ear. Time spent grazing, time spent ruminating, time spent active, time spent inactive and ear temperature was recorded per hour for every cow. Furthermore, behavioral observations were performed during 60 min per treatment on day 2, 5, 8, 22, and 34 using scan sampling with a 5-min interval according to the ethogram in Table S1 (supplementary materials). In addition to the 5-min scan sampling, if a cow interacted with the fencing system (e.g., after receiving an electrical cue from either the VF or EF) the behavioral responses of this cow were recorded during the 60-min observation period according to the ethogram in Table S2 (supplementary materials). Behavioral observations were conducted by a team of three observers. Prior to the first observation day, training was performed to standardize the use of the ethogram across observers. Results of the behavioral observations were not subjected to statistical analyses but used to evaluate whether abnormal behavior was performed that could indicate an impairment of cow welfare.

#### Milk yield, body weight, and feed intake

Milk yield was recorded at each milking session for each individual cow. Additionally, weekly milk samples (10 mL per cow) were collected during both morning and afternoon milkings in tubes containing sodium azide (5 µL) as a preservative. These samples were stored at 4°C for a maximum of one day before analysis for fat, protein, lactose, and urea content, as well as somatic cell count, by Qlip BV (Zutphen, the Netherlands). A weighted average daily milk composition was calculated based on the measured milk composition and the corresponding milk yield per milking session.

Fat- and protein-corrected milk (FPCM) was calculated weekly using the following equation: FPCM (kg/d) = (0.337 + 0.116 × fat % + 0.06 × protein %) × milk yield (kg/d) (CVB, 2016).

After leaving the milking carousel, cows passed over a GEA scale (GEA Farm Technologies Nederland BV, Deventer, the Netherlands), allowing body weight to be recorded twice daily for each cow. The scale was calibrated once per month, and if a deviation of more than 2% was detected, it was recalibrated according to the protocol of the experimental farm.

Individual concentrate feed intake from the automatic feeding stations was recorded automatically. PMR intake at the feed fence was calculated using the weighing data from the feed mixer wagon on a daily basis for each treatment, combined with the feed refusals which were weighed on three consecutive days per week for each treatment during the measurement period. The average PMR intake over these three days was considered the daily PMR intake per treatment per week and was subsequently divided by the number of cows per treatment (*n* = 16) to calculate the PMR intake per cow per day.

Fresh grass samples were collected every Tuesday during the measurement period. Sampling was conducted in the morning by walking diagonally across the pasture, cutting grass from at least 25 locations per plot at a height of 5 cm. The 25 subsamples per plot were thoroughly mixed and then stored at −20°C. At the end of the study, all samples (16 in total: four samples per week over 4 wk) were sent to Eurofins (Wageningen, the Netherlands) for analysis. The grass samples were analyzed using wet chemistry to determine dry matter content. Additional chemical components were determined using near-infrared spectroscopy (NIRS), and the net energy content of the grass was calculated according to the Dutch VEM method (Van Es 1975; Feed Unit Milk; 1,000 VEM = 6.9 MJ of NE). Furthermore, grass silage and maize silage, as individual components of the PMR, were analyzed for dry matter content and VEM. The dry matter content and VEM of concentrate feeds (both the pelleted feed and the soybean meal, dried sugar beet pulp, and lactation minerals in the PMR) were provided by the feed supplier.

Total daily feed intake per cow in the barn was calculated based on the dry matter intake of concentrates and PMR. Grass intake was estimated weekly by using the VEM system (Van Es, 1975). First, the VEM requirements of each cow were calculated based on requirements for maintenance (including an additional 930 VEM per day for daytime grazing; Hijink et al., 1987), (fat and protein corrected) milk yield, growth, and pregnancy (CVB, 2016). Next, the VEM intake from PMR and concentrates was determined for each individual cow. Finally, the dry matter intake of fresh grass per cow was estimated by calculating the difference between total VEM requirements and VEM intake in the barn, divided by the VEM content of the grass.

### Statistical analysis

Statistical analyses on data from the VF collars, milk yield, body weight and feed intake were conducted using SAS version 9.4 (SAS Institute Inc., Cary, NC). Statistical analyses on behavioral and cortisol data were conducted using RStudio (version 4.4.1). To evaluate normality of residuals, a normality test (SAS: PROC UNIVARIATE; RStudio; package “e1017”; Meyer et al., 2019) was performed where residuals were visually assessed for normality via quantile-quantile plots. The kurtosis for the success ratio and number of electrical cues was elevated. Nevertheless, normal distribution was assumed, taking into account that it was applied to a robust repeated measures mixed model with sufficient sample size. No extreme violations of model assumptions were observed after visual evaluation of residuals in quantile-quantile plots. Statistical tests were performed at a significance level of *P *≤ 0.05 for Bonferroni-corrected *P*-values. Values are presented as least squares mean (LSM) ± the maximum standard error of the mean (SEM).

To evaluate the compliance with the VF for the cows in the VF treatments, a mixed model (PROC MIXED) was used. The dependent variables were: mean number of auditory cues, mean number of electrical cues, number of successes, success ratio and confidence ratio. Means were calculated per cow per day for five periods (training period, measurement week 1–4) for these dependent variables. Independent variables were treatments (VFD, VFW), period (training, measurement week 1–4) and their interaction. Each model included period as a repeated measure with cow as the subject. Data from six days on which cows unintentionally received signals due to the system not being fully deactivated for all cows at the same time were excluded from the analysis (1 d during training, 1 d in measurement week 1, 1 d in measurement week 2, 2 d in measurement week 3 and 1 d in measurement week 4).

To evaluate the effects of fencing and grazing system on cow behavior, data from activity sensors was used in a mixed model (package ‘lme4’, RStudio). First, means were calculated from the behavioral data per cow per day for six periods (adaptation period, training period, measurement week 1–4). The dependent variables were: feeding time (min/h), rumination time (min/h), activity (min/h), and inactivity (min/h). Independent variables were treatment (VFD, VFW, EFD, EFW), period (adaptation, training, measurement week 1–4), and their interaction. Each model included period as a repeated measure with cow as the subject.

To evaluate effects of fencing and grazing system on milk and hair cortisol concentrations a mixed model (package ‘lme4’, RStudio) was used. The dependent variables were milk or hair cortisol concentrations. Independent variables were treatment (VFD, VFW, EFD, EFW), day (milk cortisol: day 2, 6, 9, 13, 16, 20, 27, and 34 or hair cortisol: start and end of study period), and their interaction. Each model included day as a repeated measure with cow as the subject.

To evaluate effects of fencing and grazing system on milk yield, body weight and feed intake, a mixed model (PROC MIXED) was used. Dependent variables were related to milk yield, body and feed intake. For each dependent variable means were calculated for the adaptation period, training period, and for each week of the measurement period, resulting in six values per cow. Independent variables were treatment (VFD, VFW, EFD, EFW), period (adaptation, training, measurement week 1–4) and their interaction. Each model included period as a repeated measure with cow as the subject.

At the beginning of day 3 of the training period, five cows were replaced with five new cows because of a mistake in the selection procedure. The 5 cows that were excluded from the study have not been included in further data analysis. On day 13 of the study, one cow was removed from the study as the VF collars were not functioning on this cow (e.g., three different collars did not yield any auditory or electrical cues over 9 d).

## Results

### Effects of virtual fencing system in different grazing systems

Cows in the VFD treatment received more auditory cues per day compared with cows in the VFW treatment over the entire study (Table 1). During training both treatments received fewer auditory cues per day compared with week 2 and tended to receive fewer auditory cues per day compared with week 3. The average number of auditory cues per day did not differ among other weeks.

**Table 1.** Mean number of auditory and electrical cues, success, success ratio and confidence ratio per cow per day of 32 dairy cows that grazed using virtual fencing and were weekly (VFW) or daily (VFD) rotated to a new plot

	Period × Treatment											*P-*value		
	Training		Week 1		Week 2		Week 3		Week 4		Ma× SEM	Treatment	Period^1^	TxP^2^
	VFW	VFD	VFW	VFD	VFW	VFD	VFW	VFD	VFW	VFD				
**Auditory cues (#/d)**	8.2	8.9	10.0	11.6	9.8	16.3	9.4	16.5	6.4	14.1	2.1	0.05	0.01	0.17
**Electrical cues (#/d)**	2.0	1.4	0.84	0.80	0.56	1.1	0.25	0.23	0.18	0.18	0.14	0.93	<0.01	<0.01
**Success (#/d)^3^**	6.2	7.4	9.2	10.8	9.2	15.1	9.1	16.2	6.2	14.0	2.05	0.05	<0.01	0.24
**Success ratio (-)^4^**	0.66	0.66	0.90	0.90	0.92	0.90	0.95	0.99	0.93	0.98	0.035	0.71	<0.01	0.64
**Confidence ratio (-)^5^**	0.25	0.32	0.39	0.46	0.37	0.58	0.40	0.61	0.28^b^	0.59^a^	0.062	0.01	<0.01	0.09

1 Five periods: training and measurement week (wk) 1–4. During the adaptation period, VF data was not yet collected and therefore not available.

2 Treatment × period interaction.

3 Success = # auditory cues − # electrical cues

4 Success ratio = success/# auditory cues.

5 Confidence ratio = (success ratio × success)/20, where success[max]=20.

a-b Values within the same period with different superscripts significantly differ (*P *< 0.05).

**Figure 2.**
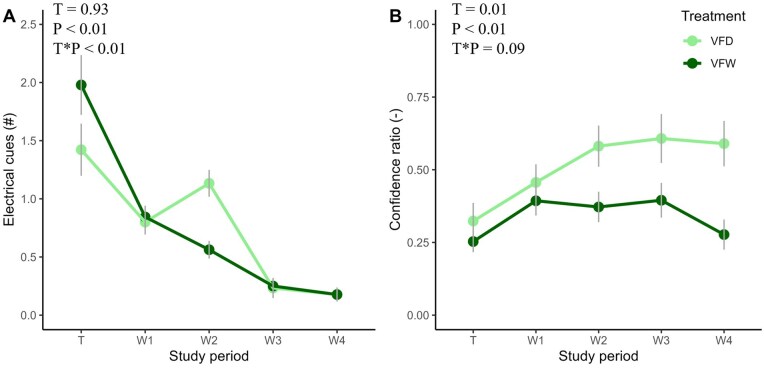
Number of electrical cues per cow per day (A) and the confidence ratio ((success ratio × success[max 20])/20) (B) for 32 dairy cows that grazed with virtual fencing (VF) and were either daily (D) or weekly (W) rotated to a new plot during the training period (T) and measurement period (week 1–4). Straight grey vertical lines indicate the standard deviation per treatment per time point.

An interaction effect between treatment and period was observed for the electrical cues, but the average number of electrical cues per day did not differ between treatments within the same week (Figure 2A). During training, cows in VFW received more electrical cues per day than during all other weeks, while cows in VFD received more electrical cues per day compared with week 1, 3, and 4. Cows in VFW tended to receive more electrical cues per day in week 1 compared with week 2 (*P *= 0.06) and received more electrical cues per day in wk 1 compared with week 3. Cows in VFD tended to receive more electrical cues per day in week 1 compared with week 4 (*P *= 0.07) and received more electrical cues per day in week 2 compared with week 3–4.

The average number of successes per day was higher for cows in VFD compared with cows in VFW during the entire study period (Table 1). During training, the average number of successes per day was lower compared with week 1–3, irrespective of treatment. Cows had a lower success ratio during training compared with all other weeks, irrespective of treatment.

For the confidence ratio, an effect of treatment, period and a tendency for an interaction between treatment and period (*P *= 0.09) was observed (Table 1). The tendency between treatment and period showed that cows in VFD had a higher confidence ratio in week 4 compared with cows in VFW (Figure 2B). No significant difference was observed between the treatments in other weeks. During training, cows in VFD had a lower confidence ratio compared with week 2–4. The confidence ratio did not differ between the different periods for cows in VFW.

### Effects of fencing system and grazing system on behavior

No aversive behaviors were observed during the study period in response to the VF or EF, based on behavioral observations. Observed behaviors after interaction with the VF or EF were turning around (body rotation of 180°) or turning sideways (body rotation of 90° to 180°), followed by walking away from the virtual or electric boundary.

**Figure 3.**
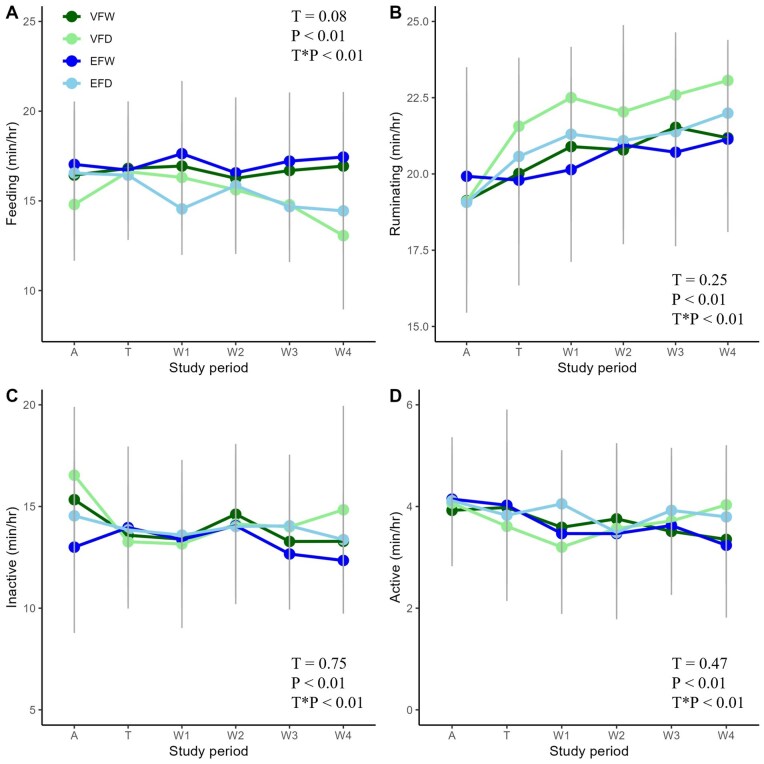
Feeding time (min/hr) (A), ruminating time (min/hr) (B), time inactive (min/h) (C) and time active (min/h) (D) for 64 dairy cows that grazed with virtual fencing (VF) or physical electric fencing (EF) and were either daily (D) or weekly (W) rotated to a new plot during different periods (P): adaptation (A), training (T) and measurement (week 1–4). Straight grey vertical lines indicate the total standard deviation across all groups per time point. T = treatment.

Feeding time, ruminating time, activity and inactivity of all cows was monitored 24 h per day and presented as average per treatment per period (Figure 3). Most time was spent feeding and ruminating, followed by inactivity and the least time was spent active over all treatments.

Both feeding time and ruminating time differed across different periods and depended on the interaction between treatment and period (Figure 3A, B). The VFD and EFD cows reduced their feeding time over the course of the study, while the feeding time remained constant in VFW and EFW. Feeding time differed between the treatments during the adaptation period, and in week 1, 3, and 4. During the adaptation period, the VFD cows spent less time feeding compared with the EFW. In week 1 and 3, the EFD cows spent less time feeding compared with the EFW cows. In week 4, the VFD and EFD cows spent less time feeding compared with the VFW and EFW cows. All cows increased their ruminating time between weeks 1 and 4. The VFD cows spent more time ruminating in week 1 compared with the EFW cows, with other treatments in between. A similar trend was shown for week 4 where the VFD cows tended to spend more time ruminating than EFW and VFW cows (*P *< 0.10).

The level of both inactivity and activity differed across the different periods and depended on the interaction between treatment and period (Figure 3C, D). The level of inactivity did not differ between treatments within the same period, and the change of inactivity over the course of the study was similar between groups. The VFW and EFW cows showed a decrease in activity throughout the study. For EFD cows, activity remained constant throughout the study, with a small decline between weeks 1 and 2. The VFD cows reduced their activity from the adaptation period to week 1, after which activity increased until week 4. Activity did not differ between treatments within the same period.

### Effects of fencing system and grazing system on cortisol concentrations in milk and hair

**Figure 4.**
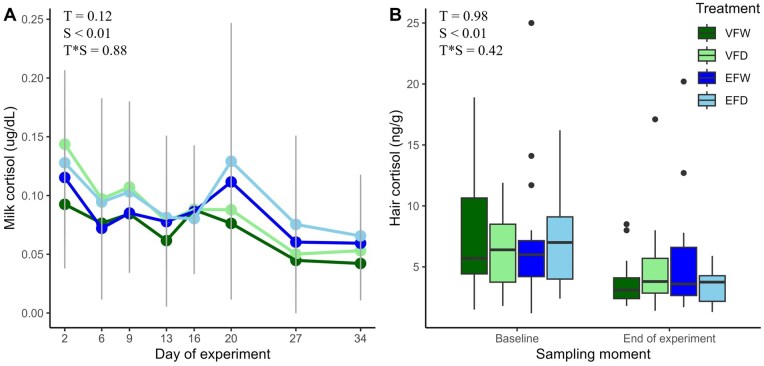
Milk cortisol (ug/dL) (A) and hair cortisol (ng/g) (B) concentrations for 64 dairy cows that grazed with virtual fencing (VF) or physical electric fencing (EF) and were either daily (D) or weekly (W) rotated to a new plot. Straight grey vertical lines indicate the total standard deviation across all groups per time point (in A). T = treatment, S = sampling moment.

Effects of fencing and grazing system on cortisol in milk were assessed, and a time effect was observed (Figure 4A). Independent of treatment, milk cortisol concentrations decreased over the course of the study. Similar results for hair cortisol concentrations were observed, where a time effect was present (Figure 4B). Independent of treatment, hair cortisol concentrations decreased between the start and end of the study.

### Effects of fencing system and grazing system on milk yield, body weight and feed intake

Table 2 shows the effects of treatment on milk yield, body weight and feed intake. The protein content in the milk of cows in VFD was higher than the protein content in the milk of cows in EFD (Table 2). The lactose content in the milk of cows in EFW was higher than the lactose content in the milk of cows in VFD. There was no effect of treatment on milk yield, FPCM, fat percentage or somatic cell count in the milk. During training, the urea content of cows in VFW was lower compared with the urea content of cows in EFD and EFW (Table 2). During this period, the urea content of cows in VFD tended to be lower compared with the urea content of cows in EFD (*P *= 0.07) and EFW (*P *= 0.07).

**Table 2.** Mean milk yield, body weight and feed intake of 64 dairy cows that grazed with virtual fencing (VF) or physical electric fencing (EF) and were either daily (D) or weekly (W) rotated to a new plot during the adaptation, training and measurement period.

	Treatment					*P-*value		
	VFW	VFD	EFW	EFD	Max SEM	Treatment	Period^1^	TxP^2^
**Milk yield**								
** Milk (kg/d)**	33.0	31.9	33.7	34.5	1.3	0.56	<0.01	0.67
** Fat-and-protein-corrected milk (kg/d)**	34.2	34.0	35.6	36.0	1.2	0.59	<0.01	0.79
** Fat content (g/100 g)**	4.5	4.6	4.5	4.5	0.01	0.74	<0.01	0.16
** Protein content (g/100 g)**	3.5^ab^	3.6^a^	3.5^ab^	3.4^b^	0.06	0.01	<0.01	0.37
** Lactose content (g/100 g)**	4.5^ab^	4.4^b^	4.5^a^	4.5^ab^	0.03	0.02	0.03	0.90
** Somatic cell count (x 1,000 cells)^4^**	56.6	71.1	45.4	55.8	1.3	0.70	0.02	0.80
** Urea (mg/dL)**	16.7	17.3	18.1	16.6	0.64	0.31	<0.01	<0.01
**Feed intake**								
** Concentrate (kg DM/d)**	5.8	5.6	5.9	6.5	0.50	0.50	<0.01	0.07
** Ration (PMR) (kg DM/d)^3^**	13.3	11.2	11.3	12.3	2.9	-	-	-
** Grass (kg DM/d)**	3.9	5.5	5.6	4.3	0.29	<0.01	<0.01	<0.01
** Total (kg DM/d)**	22.4	22.0	22.7	22.9	0.54	0.70	<0.01	0.02
**Feed efficiency**	1.5	1.5	1.5	1.5	0.02	0.63	<0.01	<0.01
**Body weight (kg)**	634	620	634	627	15.5	0.89	<0.01	<0.01

1 6 periods: adaptation, training, week 1–4. Feed intake and feed efficiency are measured during week 1–4 only (with exception of concentrate intake).

2 Treatment × period interaction

3 The PMR intake was assumed to be equal for cows within a treatment group, therefore treatment average and standard deviation instead of LSM ± SEM are shown.

4 Somatic cell count was transformed by a natural logarithm to obtain a normal distribution. Presented LSM are back transformed.

a–c Values within the same row with different superscripts differ significantly (*P *< 0.05).

An interaction effect between treatment and period was observed for grass intake (Table 2). In week 1, EFW cows had higher grass intake than cows in all other treatments. In this week, cows in VFD had a higher grass intake than cows in EFD and VFW. In week 4, cows in VFD had higher grass intake than cows in all other treatments. Also, for total feed intake, feed efficiency and body weight an interaction effect between treatment and period was observed, but the four treatments did not differ within the same weeks.

## Discussion

In this study, the application of VF in two grazing systems and the effects on behavior, cortisol concentrations, feed intake, and milk yield were investigated. Regardless of grazing system, all cows with VF showed an improvement in success, success ratio, and confidence ratio through the study, indicating that they progressively learned to respond to auditory cues to avoid receiving an electrical cue. The VF can only be effective if cows are able to understand the auditory cues and respond adequately, allowing them to avoid an electrical cue (Lee et al., 2018). A decrease in electrical cues over time could be an indication of a successful learning experience (Lomax et al., 2019). In the current study, the number of received electrical cues reduced from the training period towards the end of the study, where it stabilized in week 3 in both grazing systems.

In this study we used a training period of 4 d, and it may be questioned whether the cows were given sufficient time to learn to understand the VF. Hamidi et al. (2024) applied a training period of 12 d, whereas Verdon et al. (2021) applied a training period of 3 d with only one virtual front-fence. The individual variation in the number of electrical cues and successes during training, but also during the measurement weeks, may suggest that some cows may have benefitted from a longer training period than other cows. This was in line with other studies (Campbell et al., 2019; Lomax et al., 2019; Hamidi et al., 2024) who also reported individual variation in the VF learning process between cows. Group training was previously suggested to enhance the learning curve of individual cows (Campbell et al., 2019; Colusso et al., 2020; Verdon et al., 2020), but long-term studies towards individual differences in learning experience are required to further assess the welfare impact of VF on individuals within a herd. The required training length is likely also depending on the complexity of the environment after training. Cows introduced to a more complex environment with winding virtual boundaries after training may require a longer training period compared with cows introduced in an environment that closely resembles the training conditions, including the layout and positioning of the virtual boundaries. Further research is warranted to better understand the optimal training period duration for individual cows in VF in different grazing systems.

### Cow response to the virtual fencing system

In the current study, we observed an increase in success indicating that over time the number of auditory cues that were not followed by an electrical cue increased. In addition, the success ratio increased, indicating that the cows were able to avoid the electrical cue after receiving an auditory cue. The success ratio was comparable with a study with primiparous Fleckvieh cows (Hamidi et al., 2024), and higher than in a study with yearling Angus cows (Campbell et al., 2020) or pregnant Limousin cows (Confessore et al., 2022).

Another measure to evaluate the VF is the confidence ratio, which indicates the cows’ ability to interact with the virtual boundary without receiving an electrical cue (Hamidi et al., 2024). The confidence ratio was suggested to reflect the voluntary interaction with the VF of individual cows, as it accounts for the proportion of successful audio signals while excluding those followed by an electrical cue. The confidence ratio and its variation among cows was comparable with Hamidi et al. (2024). The confidence ratio increased over the course of the study, and this increase was most pronounced in the cows that were rotated daily to a new pasture. This indicated that cows in both grazing systems, but in particular those that were daily rotated, increased their voluntary interaction with the VF. It may be speculated that cows that were rotated daily to a new pasture were able to learn the VF more quickly because they had to adapt to changing boundaries each day unlike cows in the weekly rotating grazing system where changes occurred only once a week. However, cows that were rotated daily had access to a smaller grazing area, which may have increased their motivation to approach the virtual boundaries. This observation was also reported in other studies (Colusso et al., 2021; Grinnell et al., 2025). It may also be that the smaller area of grass increased the chance for receiving a signal in these cows, thereby enabling the cows to learn to understand the system faster. It should be noted that there was considerable variation in the confidence ratio and other calculated metrics among cows. This suggests that individual cows respond differently to the VF, highlighting the need for further research to understand how these differences affect cow health and welfare.

Differences in the success ratios and confidence ratios between different studies may be related to several factors related to the different experimental set ups. Comparison of such ratios between studies is therefore difficult. For example, our study lasted for 35 d including 31 d with VF whereas the study of Confessore et al. (2022) lasted for 16 d of which 8 d with VF. Our study showed that the cows keep on learning to deal with the VF throughout the experiment, as demonstrated by the increasing success ratio and confidence ratio towards the end of the study. The study of Campbell et al. (2020) lasted for 44 d where 20 cows had access to a pasture of 770 ha. This may have resulted in less frequent interactions with the VF, thereby resulting in a slower learning curve than in an intensive grazing system as in the current study. This is likely reflected in the differences in success ratio and confidence ratio between the studies. In addition, different breeds are known to respond differently to novel or fearful environments (Haskell et al., 2014). Age did not affect the adaptation of lactating dairy cows to a VF system, although the number of acoustic cues developed differently between younger and older cows over time (Confessore et al., 2024). Hence, it is suggested that the ratios to evaluate the understanding of the VF are mainly suitable to use for comparison of different treatments within one study rather than comparing across studies with different circumstances.

The current VF presented challenges related to human error and technological limitations. There were a few instances where the cows were removed from the pasture before the VF was properly deactivated. This occurred either because the VF was turned off too late or because the system did not respond uniformly across all animals, resulting in some cows having the VF deactivated while it remained active for some more minutes for others. Cows with an active VF often followed the cows with an already deactivated system to the barn, resulting in many undesired electrical cues. Especially in grazing systems where cows are milked daily and/or virtual boundaries are changed frequently, the VF should be able to adapt quickly to changes and (de)activation. Verdon et al. (2024) suggested that such rare or one-off events did not seem to interfere with the cow’s ability to predict and control the receipt of a pulse in the future, but further assessment of the cow’s behavior and stress responses is needed to understand the impact for cow welfare. Development of VF systems that are specifically suited for frequently-moving herds is essential to avoid welfare implications.

### Cortisol concentrations in milk and hair

Cortisol in milk and hair was measured to investigate the impact of VF and different grazing systems on stress levels. Milk cortisol is considered as an indicator for stress on the short term (hours) (Gellrich et al., 2015), and hair cortisol is considered to be an indicator of stress on the longer term (weeks to months) (Heimbürge et al., 2019). Milk samples were taken during morning milkings, and single milk samples therefore reflect stress levels during the night. Although individual milk cortisol samples reflect mainly short-term stress levels, the samples were taking twice weekly which allows to detect patterns in milk cortisol concentrations over time, thereby providing an indication of stress levels across the mid-term period (Pošćić et al., 2017). Both milk and hair cortisol concentrations were within normal ranges in the current study (Burnett et al., 2014; Fuchs et al., 2024), and milk and hair cortisol did not differ across the treatments. However, regardless of treatment, milk and hair cortisol concentrations decreased between the start and end of the study. A similar decrease in milk cortisol concentrations in dairy cows (Fuchs et al., 2024) and hair cortisol concentrations in beef cows (Jeffus et al., 2025) was reported when comparing VF and EF. Angus bulls showed a decrease in fecal cortisol concentrations after 4 wk within VF (Campbell et al., 2020). It is not fully clear what caused the decrease in cortisol concentrations. Regrouping at the start of the study may have partly contributed to the higher milk cortisol concentrations at the start of the study compared with the end (Mazer et al., 2020; Denham and Adams Progar, 2023). The decrease in cortisol concentrations it is likely not related to lactation stage of the cows, as (Fukasawa et al., 2008) showed that the lactation stage only affected milk cortisol concentrations in the first 90 d after calving. The cows in the current study were beyond 90 d in lactation.

The decrease in cortisol concentrations may be affected by housing and/or season. The housing system may affect cortisol concentrations, as a previous study of Ghassemi Nejad et al. (2021) observed a decrease in hair cortisol for cows that grazed 24 h a day compared with cows that were housed indoors. In contrast, a previous study reported an increase in hair cortisol concentrations in 83 dairy cows during the transition from winter housing to summer grazing in high mountain conditions (Comin et al., 2011). It was suggested that this rise was related to the hypothalamic–pituitary–adrenal axis response to the change from indoor winter housing to high mountain conditions. In line with this, Uetake et al. (2018) observed that the hair cortisol concentrations of lactating cows increased towards summer and this increase was most pronounced in a cold climate compared with a warmer climate. An increase in cortisol concentrations in early summer was not observed in the current study, but this could also be related to the short time window of sampling of 5 wk. In addition, the moderate Dutch summer climate was not expected to affect cortisol concentrations significantly, as only a few hot days (above 25°C) were observed, and the cows were housed indoors during the night. It may therefore be suggested that access to pasture (compared with indoor housing) had more impact on the reduction of cortisol concentrations than the type of grazing or fencing system. However, this study was only conducted over a period of a few weeks, and more research on the long-term effects of VF on the cortisol concentrations of cows is needed to better understand the impact of VF on cow welfare.

### Cow behavior

The behavior of the cows differed between treatments over the course of the study, and was mainly affected by the grazing system. Cows that were rotated daily to a new pasture reduced their feeding time over the course of the study, whereas feeding time remained constant in cows that were rotated weekly to a new pasture. This was in line with Farrell et al. (2024), who observed that grazing time decreased with more frequent pasture allocations. Cows that were rotated daily had daily availability of fresh grass and were likely able to take larger bites of the grass. As feed intake was similar between treatments, the cows that were daily rotated likely required less time to achieve the desired feed intake compared with the cows that were rotated weekly (Pulido and Leaver, 2003). The feeding time may also have been affected by differences in grass availability between pastures, as the four treatments grazed over two pastures, where grass availability was somewhat higher for the pasture where the VFD and EFW cows grazed.

Differences in ruminating time were small between treatments, but a gradual increase in ruminating time occurred for all cows over the course of the study, regardless of treatment. The increase in ruminating time likely indicated that all cows adapted their digestion to the fresh, fiber-rich grass (Abrahamse et al., 2009; Iqbal et al., 2022). Ruminating time did not seem to be affected by the fencing or grazing system in the current study. (Verdon et al., 2020) investigated that behavior and welfare of cows in the days following implementation of VF did not differ from cows in EF, although some disruptions in behavioral time budget including increased ruminating time and decreased grazing time were found between day 4 and 6 after implementation of VF. Still, feeding behavior remains an important indicator to monitor cow welfare (Nielsen et al., 2016), especially in VFs as they can be applied in different complexities.

Activity was similar between treatments at the start of the study, but differences emerged towards the end of the study between the grazing systems. The VFD cows increased their activity in week 2 and 3 compared with the VFW and EFW cows. The increased activity in the VFD cows is likely related to the daily access to a new plot as frequent allocation to a new plot has been found to affect behavioral patterns of cows (Farrell et al., 2024). The increased activity in the VFD cows could be related to the higher confidence ratio in these cows compared with VFW cows. A higher confidence ratio was suggested to indicate that cows increased their voluntary interaction with the VF and were therefore more likely to graze near the virtual boundary (Hamidi et al., 2024). This was in accordance with Staahltoft et al. (2023) and Harland et al. (2025), who showed that activity was positively correlated with the number of auditory cues from the VF. In our study, no difference in inactivity was observed between VF and EF within periods, but lying time was not measured. A previous study reported reduced total daily lying time in VF compared with EF (Campbell et al., 2019), suggesting that differences in specific resting behaviors may exist even when overall inactivity appears similar. Overall, behavior of cows seemed to be more affected by the grazing system than the fencing system in the current study.

### Feed intake and milk yield

Feed intake variables were similar between the four treatments. In a previous study, grass intake was higher for cows with EF compared with when the same cows grazed on a pasture with one VF boundary (Langworthy et al., 2021). However, this difference was compensated by a higher concentrate intake, resulting in no difference in energy intake. To better understand the differences in feed intake between cows with VF and EF, more research is required, but there are no indications that there are major differences to be found.

Milk yield, FPCM, milk fat content, and cow body weight did not differ between the four treatments, which was expected and in line with a previous study (Verdon et al., 2021). However, VFD cows had a slightly higher protein content in milk compared with EFD cows, and EFW cows had a slightly higher lactose content in milk compared with VFD cows throughout the study. The higher protein content in milk of VFD may indicate differences in available amino acids for milk protein synthesis (originating from rumen undegradable protein + rumen microbial protein) (Schingoethe, 1996; Osorio et al., 2016), resulting from differences in grass quality or percentage of fresh grass in the ration. The higher lactose content in milk in EFW cows is likely related to a higher mammary glucose uptake in these cows, but it is unclear how the actual milk lactose synthesis is affected by diet (Osorio et al., 2016). However, as glucose is a precursor for milk lactose (Lemosquet et al., 2009), and glucose is formed out of propionic acid in the rumen, dietary differences (grass quality, composition of the ration) could have influenced milk lactose content. These differences in milk composition were most likely affected by differences in grass availability between the different pastures.

This study shows that daily and weekly rotational grazing can be applied using VF in lactating dairy cows under intensive grazing conditions. Further research under various environmental conditions is required to investigate the longer-term effects of rotational grazing on cow welfare and productivity, especially given the fact that this study was conducted without replicates. When rotational grazing can be applied using VF without negative effects on cow welfare and production, it can be a promising technology to enhance rotational grazing under various conditions while decreasing labor intensity and increasing grazing efficiency. Moreover, other complex applications of VF, such as fencing out small areas to enhance biodiversity are being applied (Wätzold et al., 2024), but effects on animal welfare should be further investigated before on-farm application expands.

## Conclusion

The study showed no differences between cows in VF or EF in behavior, cortisol concentrations in hair and milk, and milk yield parameters. Regardless of grazing system, an increase in success, success ratio, and confidence ratio was observed for all cows with VF during the study, indicating that they progressively learned to use the auditory cues to avoid electrical cues. Cows that rotated daily to a new pasture within the VF had higher success and confidence ratios compared with cows that rotated weekly, indicating that more frequent shifts of the virtual boundaries enhanced learning ability and confidence in the VF. Outdoor access appeared to reduce stress levels in cows, regardless of the fencing system used.

## Supplementary Material

skaf363_Supplementary_Data

## Abbreviations

EF: physical electric fencing
EFD: electric fencing with daily rotation
EFW: electric fencing with weekly rotation
FPCM: fat- and protein-corrected milk
GNSS: global navigation satellite system
LSM: least squares mean
PMR: partial mixed ration
SEM: standard error of the mean
VF: virtual fencing system
VFD: virtual fencing with daily rotation
VFW: virtual fencing with weekly rotation
