# Supplementary material for: Effects of virtual fencing on behavior, cortisol concentrations, feed intake, and milk yield of lactating dairy cows in different grazing systems
Source: J Anim Sci. 2025 Oct 22;103:skaf363. doi: 10.1093/jas/skaf363 (PMC12629541; doi:10.1093/jas/skaf363)
Supplement: skaf363_Supplementary_Data [file skaf363_supplementary_data.zip › Supplementary material.docx]

**Supplementary material**

**Table S1** Ethogram for 5-minute scan sampling of cow behavior in the pasture

| Behavior | Description |
| --- | --- |
| Walking | Move forward with one leg at a time in an even stride. The movement continues for more than one body length. |
| Trotting | Move forward at a speed faster than walking. The head is held up. The movement continues for more than one body length. |
| Running | Move quickly forward with a maximum of 2 legs on the ground at the same time. |
| Lying | Rest with the whole body on the ground. |
| Grazing | Head bent toward the ground and frequently taking bites of grass. |
| Drinking | Head bent into the water trough and taking in water. |
| Other | Other behavior. |

**Table S2** Ethogram for recording cow behavior after an observed interactions with the (virtual) fencing of cows in the pasture

| Behavior | Description |
| --- | --- |
| Bucking | Both hind legs off the ground and stretched backward. Front legs remain on the ground. |
| Head shaking | Vigorous movement of the head and/or neck from left to right. |
| Vocalizing | A fearful call with a low tone after receiving a stimulus. |
| Escaping | Crossing the (virtual) boundary for at least 5 meters. |
| Turning sideways | Body rotation of 90 to 180°, so the animal is (almost) parallel to the (virtual) fence. |
| Turning around | Body rotation of about 180°, so the cow turns away from the (virtual) fence. |
| 360° turning | Full body rotation of 360° or more. |
| Other | Other behavior. |
